# Decreased Resting-State Interhemispheric Functional Connectivity in Medication-Free Obsessive-Compulsive Disorder

**DOI:** 10.3389/fpsyt.2020.559729

**Published:** 2020-09-15

**Authors:** Cuicui Jia, Yangpan Ou, Yunhui Chen, Ping Li, Dan Lv, Ru Yang, Zhaoxi Zhong, Lei Sun, Yuhua Wang, Guangfeng Zhang, Hong Guo, Zhenghai Sun, Wei Wang, Yefu Wang, Xiaoping Wang, Wenbin Guo

**Affiliations:** ^1^ Department of Psychiatry, Qiqihar Medical University, Qiqihar, China; ^2^ National Clinical Research Center for Mental Disorders, and Department of Psychiatry, The Second Xiangya Hospital of Central South University, Changsha, China; ^3^ Department of Radiology, The Second Xiangya Hospital of Central South University, Changsha, China; ^4^ Henan Key Lab of Biological Psychiatry, The Second Affiliated Hospital of Xinxiang Medical University, Xinxiang, China; ^5^ Department of Radiology, The Third Affiliated Hospital of Qiqihar Medical University, Qiqihar, China; ^6^ Department of Radiology, The First Hospital of Qiqihar, Qiqihar, China; ^7^ Department of Library, Qiqihar Medical University, Qiqihar, China

**Keywords:** obsessive-compulsive disorder, voxel-mirrored homotopic connectivity, support vector machine, functional magnetic resonance imaging, resting-state

## Abstract

**Objective:**

Decreased homotopic connectivity of brain networks such as the cortico-striato-thalamo-cortical (CSTC) circuits may contribute to the pathophysiology of obsessive-compulsive disorder (OCD). However, little is known about interhemispheric functional connectivity (FC) at rest in OCD. In this study, the voxel-mirrored homotopic connectivity (VMHC) method was applied to explore interhemispheric coordination at rest in OCD.

**Methods:**

Forty medication-free patients with OCD and 38 sex-, age-, and education level-matched healthy controls (HCs) underwent a resting-state functional magnetic resonance imaging. The VMHC and support vector machine (SVM) methods were used to analyze the data.

**Results:**

Patients with OCD had remarkably decreased VMHC values in the orbitofrontal cortex, thalamus, middle occipital gyrus, and precentral and postcentral gyri compared with HCs. A combination of the VMHC values in the thalamus and postcentral gyrus could optimally distinguish patients with OCD from HCs.

**Conclusions:**

Our findings highlight the contribution of decreased interhemispheric FC within and outside the CSTC circuits in OCD and provide evidence to the pathophysiology of OCD.

## Introduction

The features of obsessive-compulsive disorder (OCD) are persistent and intrusive thoughts, impulses, images, and repetitive behaviors (1). This chronic neuropsychiatric disorder has a lifetime prevalence of 2% to 3% (2). As one of the top ten disabling diseases, OCD leads to social disability, functional impairment, and even suicide. OCD imposes considerable financial burden and misery to families. The involvement of structural and functional abnormalities in the areas of the cortico-striato-thalamo-cortical (CSTC) circuits, which include the orbitofrontal cortex (OFC), dorsolateral prefrontal cortex, anterior cingulate cortex, striatum, and thalamus, is among the most examined networks in OCD (3–5). However, other brain regions outside the CSTC circuits, such as the occipital, parietal, and cerebellar cortices, are also involved in OCD (6–8).

As the largest commissure of white matter, the corpus callosum (CC) plays a crucial role in cognitive processes and interhemispheric communication (9). Decreased fractional anisotropy values (10) and microstructural abnormalities in the CC (11) have been reported in OCD, which may affect interhemispheric functional interactions related to obsessions and compulsions (12). Functional interhemispheric coordination between the cerebral hemispheres may be an important aspect of brain function (13). Nevertheless, little is known about alterations in functional interhemispheric coordination in OCD.

As a basic characteristic of the brain’s intrinsic functional architecture, functional homotopy reﬂects high degree of synchrony in spontaneous activity between geometrically corresponding (i.e., homotopic) regions in each hemisphere, and can be investigated with the resting-state functional connectivity (FC) method (14). A voxel-mirrored homotopic connectivity (VMHC) approach explores the resting-state FC between the time series for each voxel in one hemisphere and that of its corresponding voxel in the opposite hemisphere (14) and is designed to measure interhemispheric coordination (15). This method has been well applied in neuropsychiatric disorders (16–20). Previous studies used the VMHC approach to explore interhemispheric patterns in patients with OCD at rest and found reduced VMHC values within and outside the CSTC circuits (21, 22). Their findings indicated that the integrity of interhemispheric cooperation was impaired in OCD. However, most patients with OCD in the previous studies were on medications that might affect brain connectivity at rest (21–24). Whether altered interhemispheric coordination can be applied as a potential image marker to determine OCD from healthy controls (HCs) remains unclear.

In this study, we investigated interhemispheric FC at rest in a relatively large number of medication-free patients with OCD using the VMHC method. We hypothesized that patients with OCD would exhibit decreased VMHC within and outside the CSTC circuits based on previous studies (21, 22). We further explored the correlations between altered VMHC values and clinical variables in patients with OCD. Machine-learning techniques have received increasing attention and are used to identify potential neuroimaging biomarkers for early diagnosis in psychiatric disorders (25). Support vector machine (SVM) is one of the machine-learning techniques with higher precision and accuracy. SVM is effective to define a set of information and features from different brain regions that can classify patients and HCs using neuroimaging data such as functional magnetic resonance imaging (fMRI) data (25). Therefore, the present research applied SVM to detect whether abnormal VMHC could differentiate patients with OCD from HCs.

## Materials and Methods

### Participants

Forty patients with OCD were recruited from the Fourth Affiliated Hospital of Qiqihar Medical University, and Qiqihar Mental Health Center, China. Diagnosis of OCD was confirmed according to the Structured Clinical Interview for DMS-IV (SCID), patient version (26). Yale-Brown Obsessive-Compulsive Scale (Y-BOCS), 17-item Hamilton Depression Rating Scale (HAMD), and Hamilton Anxiety Rating Scale (HAMA) were used to assess the severity of clinical symptoms of OCD. Eighteen patients were drug-naive, and twenty-two had a history of antidepressants, antipsychotics or anti-obsessive medication. No patients had taken any kind of psychotropic medication for at least four weeks before the recruitment. Thirty-eight sex-, age-, and education level-matched HCs were enrolled from the local community using the SCID, non-patient version (27). Patients with OCD and HCs had the same inclusion criteria: (1) 16 to 50 years of age; (2) right-handedness; (3) no neurological disorder; (4) no severe medical disorder; (5) no substance/alcohol abuse or nicotine/caffeine dependence; and (6) no contraindication for MRI scan. HCs were excluded if they had a first-degree relative with any psychiatric disorders.

Ethical approval was obtained from the Research Ethics Committee of Qiqihar Medical University. All subjects were informed about the full description of procedures and provided a written informed consent.

### MRI Data Acquisition

Brain images were implemented on a 3.0-Tesla GE 750 Signa-HDX scanner (General Electric Healthcare, Waukesha, Wisconsin) with a standard head coil (12-channel) at the Third Affiliated Hospital of Qiqihar Medical University. All individuals were instructed to lie still with closed eyes and minimize head movement. An echo-planar imaging (EPI) sequence was used to acquire resting-state fMRI with the following parameters: 33 axial slices, 2000-ms TR, 30-ms TE, 3.5/0.6-mm thickness/gap, 90° flip angle, 200 × 200-mm^2^ field of view, 64 × 64 data matrix, and 240 volumes in total.

### Data Processing

The imaging data were processed through the Data Processing & Analysis for Brain Imaging (DPABI) software (28). The preprocessing procedures were as follows: the first 10 volumes were discarded. A slice-timing correction was applied. After that, the images were spatially normalized to a standard Montreal Neurological Institute (MNI) space and resembled to 3 × 3 × 3 mm^3^. Subsequently, the processed images were spatially smoothed using an isotropic Gaussian kernel of 4 × 4 × 4 mm^3^ full width at half maximum (FWHM). The temporal band-pass filtering (0.01–0.08 Hz) was conducted to reduce the confounding effect of high-frequency physiological noise and low-frequency drifts. White matter, cerebrospinal fluid time course, and 24 head motion parameters were regressed out as the nuisance covariates. Mean framewise displacement (FD) for each participant was calculated. Scrubbing was applied with a FD which indexes volume-to-volume changes in head position using a threshold of 0.2 together with one preceding and two subsequent volumes (29).

#### Interhemispheric Correlation

VMHC computation was also processed by using the DPABI software (28). The homotopic resting-state FC of the individuals was computed as the Pearson correlations between the residual time series of each voxel in one hemisphere and that of its opposite hemisphere. Then, the coefficients were Fisher *z*-transformed, and VMHC maps were generated with the resultant values. The mean VMHC was extracted from the voxels that make up the clusters.

#### Statistical Analyses

The demographic and clinical characteristics of the two groups were conducted with two-sample *t*-tests or a χ^2^ test using SPSS version 23.0 (SPSS Inc., Chicago, IL, USA).

The mean FD and age were subsequently utilized as covariates for voxel-wise two-sample *t*-tests between drug-naive patients with OCD and HCs to detect VMHC differences. The significance level corrected by the Gaussian random field (GRF) theory was *p* < 0.05. To minimize the confounding effects of depressive and anxiety symptoms on the present results, we repeated voxel-wise two-sample *t*-tests using the mean FD, age, HAMD scores, and HAMA scores as covariates.

Pearson correlations were evaluated between abnormal VMHC and clinical symptoms, i.e., total, obsession, and compulsion scores in the Y-BOCS, HAMA scores, and HAMD scores in patients with OCD. The Bonferroni-corrected significance level was *p* < 0.05.

#### SVM Analysis

SVM analysis was conducted using the LIBSVM software in MATLAB (http://www.csie.ntu.edu.tw/cjlin/libsvm/). This exploratory analysis examined whether abnormal VMHC could be used to differentiate patients with OCD from HCs. SVM classifiers can separate the individuals into two classes through a decision boundary, which is as far as the closest points from each of the sample data according to a function of selecting kernel. To minimize the problem of overfitting or underfitting, exploratory SVM analysis is conducted in the LIBSVM software with default parameters. A “leave-one-out” cross-validation approach was used to obtain the highest values for specificity and sensitivity. A permutation test was applied to validate the SVM results, which ran 10,000 times for each sample (OCD/HCs). Then, a global accuracy could be obtained for each sample.

## Results

### Demographic Characteristics of Subjects

The 40 patients with OCD comprised 27 men and 13 women. The 38 HCs comprised 25 men and 13 women. Patients and HCs showed no significant between-group differences in age (*t* = 0.05, *p* = 0.71), gender (*X^2^* = 0.32, *p* = 1.00), education level (*t* = 0.50, *p* = 0.83), and FD (*t* = 1.25, *p* = 0.13). Between-group differences were found in the clinical characteristics of Y-BOCS (*t* = 25.27, *p* < 0.01), HAMA (*t* = 9.00, *p* < 0.01), and HAMD (*t* = 9.04, *p* < 0.01). Details are shown in **Supplementary Material Table S1**.

### VMHC Differences

**Figure 1:**
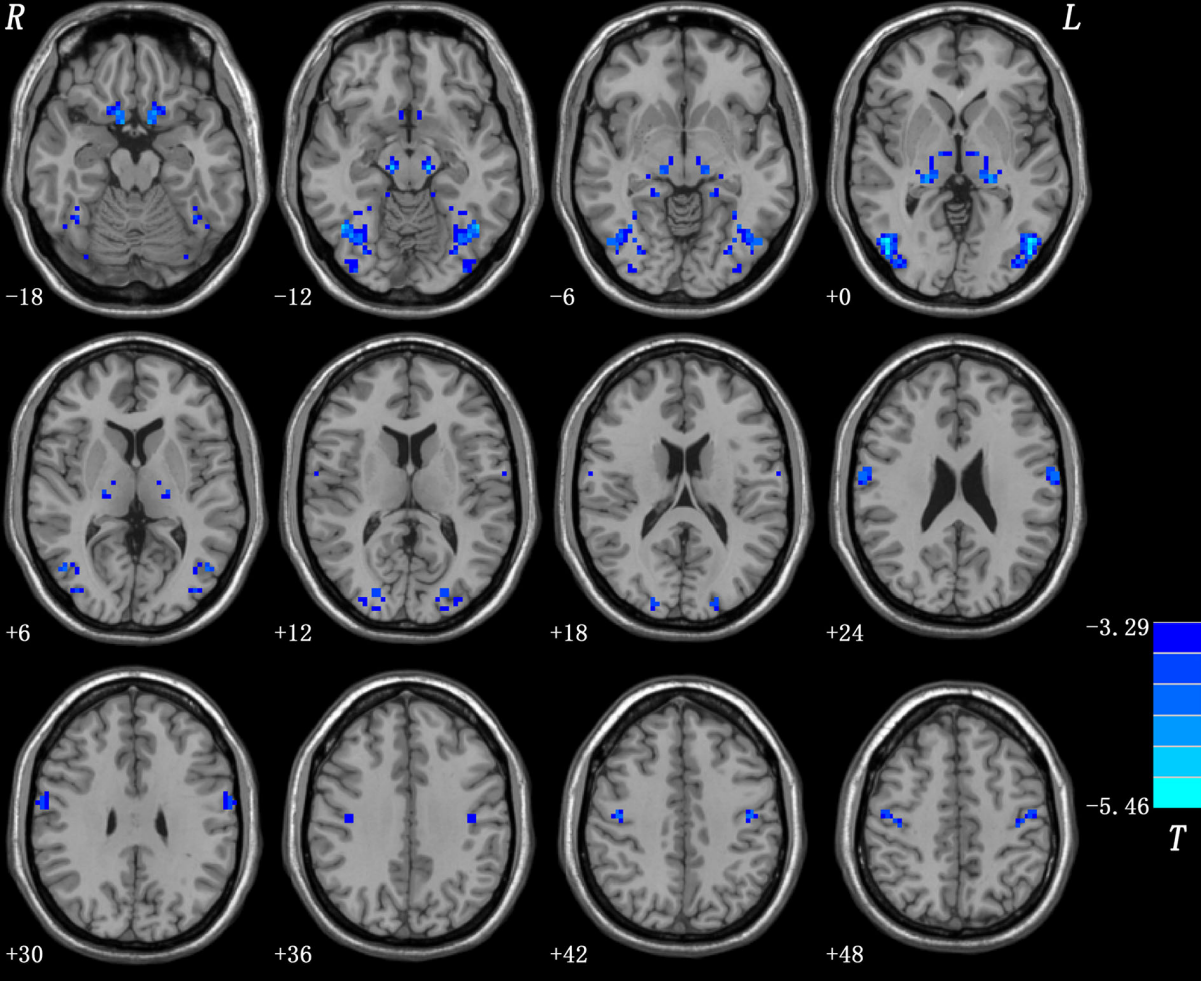
Brain regions with decreased VMHC in patients with OCD. Blue denotes decreased VMHC. Color bars indicate *t*-values of voxel-based two-sample *t*-tests (*p* < 0.05, GRF-corrected).

**Figure 2:**
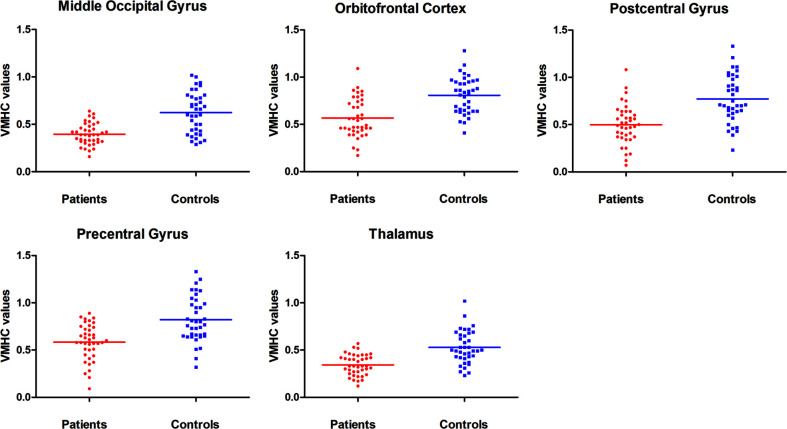
Scatter plots for the significant clusters of samples. VMHC, voxel-mirrored homotopic connectivity.

Patients with OCD had remarkably decreased VMHC values in the OFC, thalamus, middle occipital gyrus, and precentral and postcentral gyri compared with HCs (**Table 1** and **Figure 1**). No VMHC was increased in the patients relative to the controls. These results were similar using the mean FD, age, HAMD scores, and HAMA scores as covariates (**Table S2**). Scatter plots for the significant clusters of samples (OCD/HCs) were showed in **Figure 2**.

**Table 1:** Decreased VMHC in patients with OCD.

Cluster location	Peak (MNI)			Number of voxels	*T* value
	x	y	z		
Orbitofrontal Cortex	± 9	9	−18	64	−4.5406
Thalamus	±12	−21	−12	152	−4.9082
Middle Occipital Gyrus	±48	−69	0	506	−5.2791
Postcentral Gyrus	±63	−6	27	92	−4.8418
Precentral Gyrus	±42	−15	39	64	−4.5126

MNI, Montreal Neurological Institute; VMHC, voxel-mirrored homotopic connectivity.

The significance level was set at p< 0.05 corrected by the Gaussian random field (GRF) theory (voxel significance: p < 0.001, cluster significance: p < 0.05) for multiple comparisons.

### Correlation Analysis

No correlations were found between abnormal VMHC values and clinical variables (i.e., total, obsession, and compulsion scores in the Y-BOCS, HAMA and HAMD scores) in patients with OCD at Bonferroni-corrected *p* < 0.05 level.

### SVM Results

**Figure 3:**
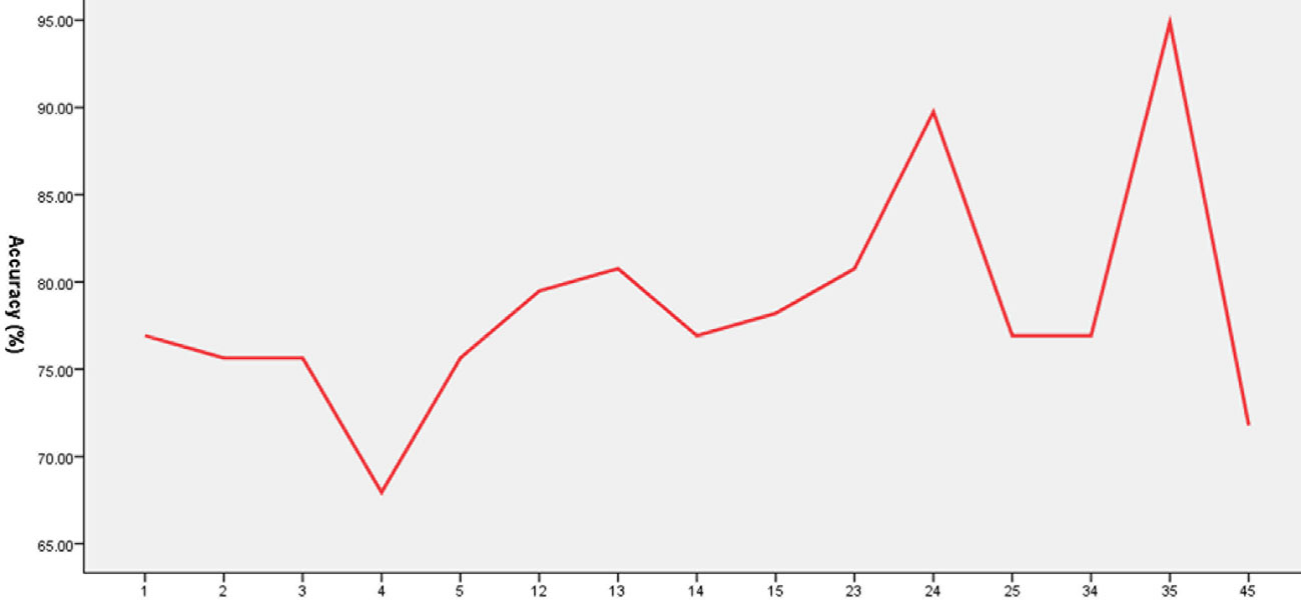
Accuracy (%) of abnormal VMHC in single or combined brain regions to discriminate patients from healthy controls. 1, Middle occipital gyrus; 2, orbitofrontal cortex; 3, postcentral gyrus; 4, precentral gyrus; 5, thalamus; 12, middle occipital gyrus and orbitofrontal cortex; 13, middle occipital gyrus and postcentral gyrus; 14, middle occipital gyrus and precentral gyrus; 15, middle occipital gyrus and thalamus; 23, orbitofrontal cortex and postcentral gyrus; 24, orbitofrontal cortex and precentral gyrus; 25, orbitofrontal cortex and thalamus; 34, postcentral gyrus and precentral gyrus; 35, postcentral gyrus and thalamus; 45, precentral gyrus and thalamus.

**Figure 4:**
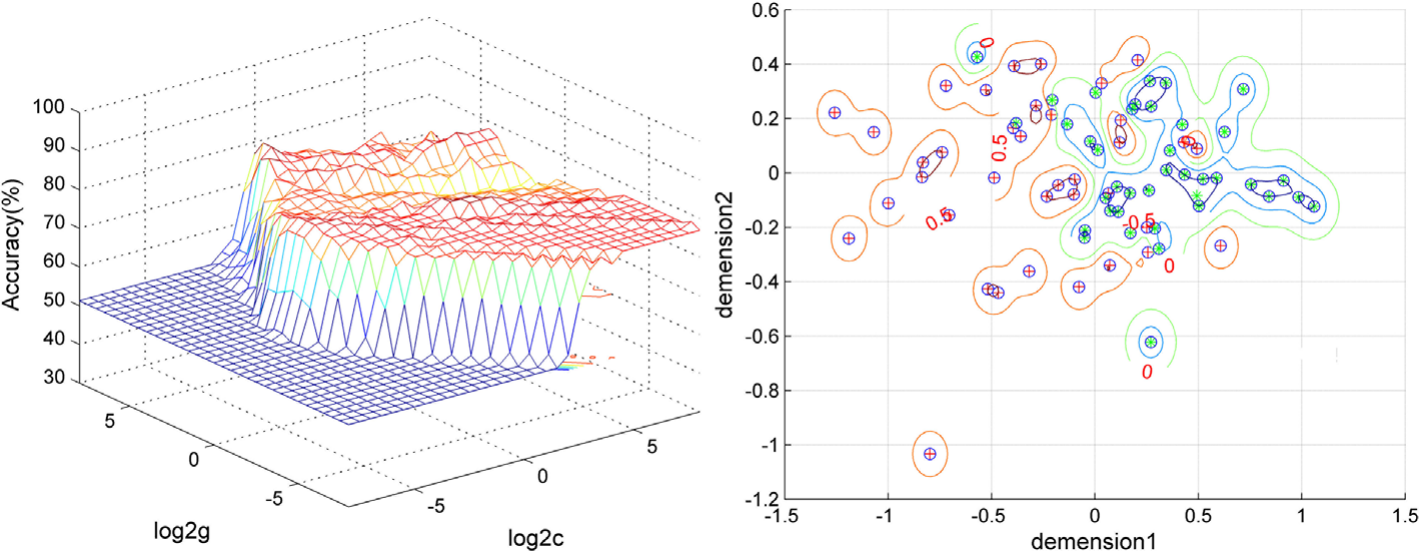
Visualization of the SVM classification using the combination of the VMHC values in the thalamus and postcentral gyrus. Left: 3D visualization of the SVM parameter selection result (Best c, 1; Best g, 128). Right: visualization of the classification with the combination of the VMHC values in the thalamus and postcentral gyrus. Different colors of the contour lines indicate different classification accuracies by using different combinations of the VMHC values in the thalamus and postcentral gyrus. Dimension 1, the VMHC values in the postcentral gyrus; dimension 2, the VMHC values in the thalamus.

Five brain regions (1 = middle occipital gyrus, 2 = orbitofrontal cortex, 3 = postcentral gyrus, 4 = precentral gyrus, and 5 = thalamus) in patients with OCD had decreased VMHC values. Exploratory SVM analysis was conducted using the VMHCs of these five brain regions and their pairwise combinations. Their classification accuracies were as follows: 1 = 76.92% (60/78), 2 = 75.64% (59/78), 3 = 75.64% (59/78), 4 = 67.95% (53/78), 5 = 75.64% (59/78), 1 and 2 = 79.49% (62/78), 1 and 3 = 80.77% (63/78), 1 and 4 = 76.92% (60/78), 1 and 5 = 78.21% (61/78), 2 and 3 = 80.77% (63/78), 2 and 4 = 89.74% (70/78), 2 and 5 = 76.92% (60/78), 3 and 4 = 76.92% (60/78), 3 and 5 = 94.87% (74/78), and 4 and 5 = 71.79% (56/78).The combination of the VMHC values of 3 and 5 (**Figures 3** and **4**) could optimally discriminate the patients with OCD from the HCs with accuracy, sensitivity, and specificity rates of 94.87%, 95.00%, and 94.74%, respectively.

To validate the SVM results. A permutation test showed that the global accuracy was 0.9562 (*p* < 0.001) for discriminating patients with OCD from HCs by using the combination of the VMHC values of 3 and 5.

A feature selection was conducted to validate the SVM results as follows. First, the accuracy was 82.05% (64/78) using the combination of abnormal VMHC values in five brain regions for classification. Then, the VMHC values in one brain region were removed and the VMHC values in the remaining four brain regions were combined for classification. If the accuracy increased, it means that the VMHC values in this brain region could be removed; if the accuracy decreased, it means that the VMHC values in this brain region should be retained. Next, the VMHC values in another brain region were removed, the VMHC values in the remaining three brain regions were combined for classification, and the above process was repeated. Finally, the combination of the VMHC values in the postcentral gyrus and thalamus (3 and 5, **Figures 3**, **4**) could optimally discriminate the patients from the HCs with an optimal accuracy of 94.87%.

**Figure 5:**
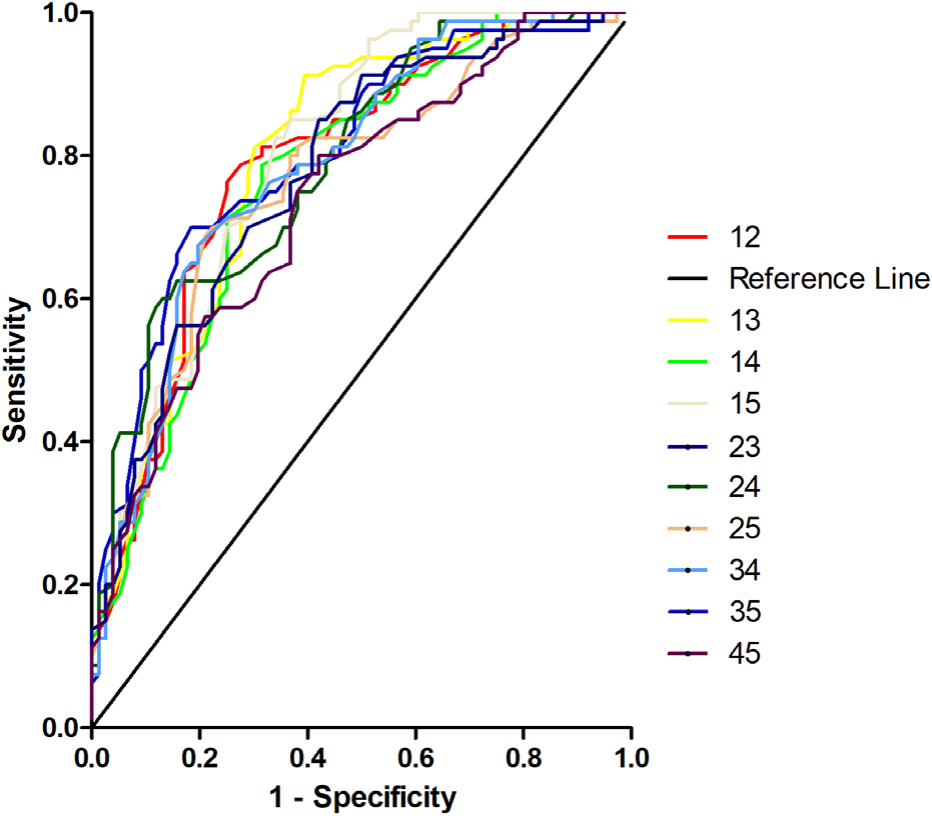
Receiver operating characteristic curves (ROC) for discrimination patients with obsessive-compulsive disorder from healthy controls using different combinations of voxel-mirrored homotopic connectivity values in five brain regions. 12, Middle occipital gyrus and orbitofrontal cortex; 13, middle occipital gyrus and postcentral gyrus; 14, middle occipital gyrus and precentral gyrus; 15, middle occipital gyrus and thalamus; 23, orbitofrontal cortex and postcentral gyrus; 24, orbitofrontal cortex and precentral gyrus; 25, orbitofrontal cortex and thalamus; 34, postcentral gyrus and precentral gyrus; 35, postcentral gyrus and thalamus; 45, precentral gyrus and thalamus.

Different combinations of abnormal VMHC values in five brain regions were examined to validate the SVM results using the receiver operating characteristic curve (ROC) (**Figure 5**). The areas under the curve (AUC) were as follows: 1 and 2 = 0.7889, 1 and 3 = 0.7965, 1 and 4 = 0.7717, 1 and 5 = 0.8030, 2 and 3 = 0.7774, 2 and 4 = 0.7899, 2 and 5 = 0.7666, 3 and 4 = 0.7881, 3 and 5 = 0.8047, and 4 and 5 = 0.7387. As expected, the combination of 3 and 5 showed the highest AUC.

## Discussion

The present study explored interhemispheric FC at rest in a relatively large medication-free OCD sample. Consistent with our hypothesis, patients with OCD exhibited decreased VMHC values within (i.e., OFC and thalamus) and outside (i.e., middle occipital, precentral, and postcentral gyri) the CSTC circuits. In addition, a combination of decreased VMHC in the thalamus and postcentral gyrus could distinguish patients with OCD from HCs with high accuracy, specificity, and sensitivity.

This study discovered decreased VMHC values within the CSTC circuits, such as OFC and thalamus, in patients with OCD at rest. OFC is a key structure within the CSTC circuits and plays an important role in response inhibition, behavioral inhibition, and emotional regulation (30–32). As a central relay station (33), the thalamus transmits sensory input from the surrounding area to the cortex and manages input and output information between the striatum and cortex within the CSTC circuits (3, 34). A recent meta-analysis discovered that patients with OCD showed hypoactivation in the bilateral thalamus and medial OFC during inhibitory control (35). Decreased VMHC values in the OFC and thalamus in patients with OCD at rest were also observed in other studies (21, 22). The present and previous studies supported the disconnection between homotopic brain regions within the CSTC circuits in patients with OCD, which might lead to obstacles in the communication and integration of cognitive and emotional information in OCD (15).

Decreased VMHC outside the CSTC circuits, such as the middle occipital, precentral, and postcentral gyri, in patients with OCD at rest in our study is similar to the results of Deng et al. (21). The occipital cortex is involved in the pathophysiology of OCD because of its connection with the CSTC circuits (4, 36). Abnormalities in the occipital cortex in patients with OCD may underlie the visuospatial deficits characteristic of the disorder involved in the pathophysiology of OCD, and cognitive impairments in OCD are also underpinned by disconnectivity of specific neurocognitive networks (37). Decreased regional homogeneity (ReHo) in the occipital cortex and increased FC with the caudate nucleus have been discovered in different samples of OCD in our previous studies (36, 38). Based on our current and previous findings, we inferred that decreased ReHo and interhemispheric functional homotopy in the occipital cortex and increased FC with the CSTC circuits may work together to contribute to the pathogenesis of OCD. The precentral and postcentral gyri are important brain regions in the sensorimotor network; decreased global brain FC in the precentral or postcentral gyrus at rest has been found in patients with OCD (39), and the amplitude of the low-frequency ﬂuctuations of the precentral gyrus can be used to distinguish patients with OCD from HCs (8). In the present study, decreased interhemispheric functional homotopy in the precentral and postcentral gyri may be associated with damaged sensory-motor integration and sensory gating in OCD at rest and might contribute to the inability to suppress internally repetitive and intrusive thoughts and behaviors in patients with OCD (40, 41). Brain regions with macro- and microstructural alterations outside the CSTC circuits are also related to the pathophysiology of OCD (6–8, 11, 42–44). The present findings provide additional evidence to elucidate the pathogenesis of OCD.

The SVM classification method is a binary classification algorithm that maximizes the boundary between classes in a high dimensional space (45). The current SVM results displayed that the combination of decreased VMHC in the thalamus and postcentral gyrus could distinguish patients with OCD from HCs with optimal accuracy, specificity, and sensitivity and could be used as a potential neurobiological marker for OCD. This marker may improve diagnostic accuracy and provide a new perspective for clinical diagnosis.

This study had several limitations. First, the human brain is asymmetrical, and this asymmetry may cause bias in the present findings. However, we used a symmetrical template and smoothed the data during data processing to limit the possible effects of asymmetry. Second, gray and white matters were not assessed in the current study, and their potential influence on VMHC was unclear. Third, patients with OCD were not divided into different subtypes according to clinical symptoms. Fourth, some patients had a history of taking psychotropic medication, which might influence interhemispheric functional homotopy at rest in OCD. Fifth, resting-state FC is altered in depression, and even subclinical depressive phenomenology can modify the brain’s microstructure (46). Although only very mild symptoms were present in this cohort (but see the significant difference between HAMA and HAMD scores in the two samples), this might have impacted the present findings. To minimize the confounding effects of depressive and anxiety symptoms on the present results, we repeated voxel-wise two-sample *t*-tests using the mean FD, age, HAMD scores, and HAMA scores as covariates. Similar results were obtained. This issue indicated that depressive and anxiety symptoms had little effect on the present results. Finally, it may be circular to pick brain regions already shown difference between groups in the univariate voxel-wise analysis, and the results of SVM may be potential overfitting/unfitting due to small sample size (47). For these reasons, interpretation of the present findings should be cautious.

Despite these limitations, our findings found decreased VMHC values within and outside the CSTC circuits at rest in patients with OCD. A combination of the VMHC values of the thalamus and postcentral gyrus could be used as a discriminative feature to distinguish patients with OCD from HCs. The current study provides evidence to the pathophysiology of OCD.

## Data Availability Statement 

The original contributions presented in the study are included in the article/**Supplementary Material**; further inquiries can be directed to the corresponding authors.

## Ethics Statement

The studies involving human participants were reviewed and approved by: Ethical approval was obtained from the Research Ethics Committee of Qiqihar Medical University. The patients/participants provided their written informed consent to participate in this study.

## Author Contributions 

CJ and PL conceived, designed, and wrote the study. WG put forward effective suggestions for the revision of the article. YC, YuW, ZS, XW, RY, ZZ, GZ, HG, and WW carried out and collected the data. YO, DL, LS, and YeW analyzed the data. All authors contributed to the article and approved the submitted version.

## Funding

This study was supported by grants from Heilongjiang Natural Science Foundation of China (LH2019H064), and Project of Heilongjiang Provincial Department of Education (2018-KYYWF-0111).

## Conflict of Interest

The authors declare that the research was conducted in the absence of any commercial or financial relationships that could be construed as a potential conflict of interest.
