# Supplementary material for: Decreased Resting-State Interhemispheric Functional Connectivity in Medication-Free Obsessive-Compulsive Disorder
Source: Front Psychiatry. 2020 Sep 15;11:559729. doi: 10.3389/fpsyt.2020.559729 (PMC7522198; doi:10.3389/fpsyt.2020.559729)
Supplement: Supplementary file 1 [file DataSheet_1.docx]

Supplementary Material

# TABLES

Table S1 Demographic and clinical characteristics of participants

|  | OCD patients  (n =40) | HCs  (n = 38) | *X^2^*/*t* | *p* |
| --- | --- | --- | --- | --- |
| Age (years) | 27.28 ± 8.16 | 27.18 ± 8.33 | 0.05 | 0.71 |
| Sex (male/female) | 27/13 | 25/13 | 0.32 | 1.00 |
| Education (years) | 13.40 ± 2.87 | 13.74 ± 3.03 | -0.50 | 0.83 |
| Illness duration (months) | 66.68 ± 75.54 |  |  |  |
| Y-BOCS total score | 24.90 ± 5.73 | 1.13 ± 0.88 | 25.27 | 0.00 |
| Y-BOCS obsessive thinking | 12.85 ± 4.25 | 0.37 ± 0.49 | 17.98 | 0.00 |
| Y-BOCS compulsive behavior | 12.05 ± 4.62 | 0.74 ± 0.72 | 14.92 | 0.00 |
| HAMD | 8.05 ± 4.40 | 1.45 ± 0.95 | 9.04 | 0.00 |
| HAMA | 10.83 ± 6.55 | 1.16 ± 1.00 | 9.00 | 0.00 |
| FD | 0.04 ± 0.02 | 0.03 ± 0.01 | 1.25 | 0.13 |
| Time points scrubbed out | 1.13 ± 2.256 | 1.00 ± 2.418 | 0.25 | 0.95 |

OCD = obsessive-compulsive disorder; Y-BOCS = Yale-Brown Obsessive-Compulsive Scale; HAMD = 17-item Hamilton Depression Rating Scale; HAMA = Hamilton Anxiety Rating Scale; FD = framewise displacement. Variables of age, education, Y-BOCS total score, subscales score, HAMD score, HAMA score and FD were tested by two sample *t*-test, the results were indicated by *t* values. Categorical data such as gender was tested using a chi-squared test, the result was indicated by *X^2^*.

Table S2. Decreased VMHC in patients with OCD

| Cluster location | Peak (MNI) | | | Number of voxels | *T* value |
| --- | --- | --- | --- | --- | --- |
|  | x | y | z |  |  |
| Orbitofrontal Cortex | ±9 | 9 | -18 | 64 | -4.6230 |
| Thalamus | ±12 | -21 | -12 | 158 | -4.8767 |
| Middle Occipital Gyrus | ±48 | -78 | 0 | 458 | -5.4041 |
| Postcentral Gyrus | ±63 | -6 | 27 | 94 | -4.9084 |
| Precentral Gyrus | ±42 | -15 | 39 | 70 | -4.4929 |

MNI = Montreal Neurological Institute; VMHC = voxel-mirrored homotopic connectivity. The results were obtained using the mean FD, age, HAMD scores, and HAMA scores as covariates. The significance level was set at *p* < 0.05 corrected by the Gaussian random field (GRF) theory (voxel significance: *p* < 0.001, cluster significance: *p* < 0.05) for multiple comparisons.
